# Optimization of Fast Non-Local Means Noise Reduction Algorithm Parameter in Computed Tomographic Phantom Images Using 3D Printing Technology

**DOI:** 10.3390/diagnostics14151589

**Published:** 2024-07-23

**Authors:** Hajin Kim, Sewon Lim, Minji Park, Kyuseok Kim, Seong-Hyeon Kang, Youngjin Lee

**Affiliations:** 1 Department of Health Science, General Graduate School of Gachon University, 191, Hambakmoe-ro, Yeonsu-gu, Incheon 21936, Republic of Korea; happida3@gachon.ac.kr (H.K.); tpdnjs728@gachon.ac.kr (S.L.); mu05430@gachon.ac.kr (M.P.); 2 Department of Biomedical Engineering, Eulji University, 553, Sanseong-daero, Sujeong-gu, Seongnam-si 13135, Republic of Korea; kskim502@eulji.ac.kr; 3 Department of Radiological Science, Gachon University, 191, Hambakmoe-ro, Yeonsu-gu, Incheon 21936, Republic of Korea

**Keywords:** computed tomography, fast non-local means approach, 3D printing technology, self-produced phantom, quantitative evaluation of image quality

## Abstract

Noise in computed tomography (CT) is inevitably generated, which lowers the accuracy of disease diagnosis. The non-local means approach, a software technique for reducing noise, is widely used in medical imaging. In this study, we propose a noise reduction algorithm based on fast non-local means (FNLMs) and apply it to CT images of a phantom created using 3D printing technology. The self-produced phantom was manufactured using filaments with similar density to human brain tissues. To quantitatively evaluate image quality, the contrast-to-noise ratio (CNR), coefficient of variation (COV), and normalized noise power spectrum (NNPS) were calculated. The results demonstrate that the optimized smoothing factors of FNLMs are 0.08, 0.16, 0.22, 0.25, and 0.32 at 0.001, 0.005, 0.01, 0.05, and 0.1 of noise intensities, respectively. In addition, we compared the optimized FNLMs with noisy, local filters and total variation algorithms. As a result, FNLMs showed superior performance compared to various denoising techniques. Particularly, comparing the optimized FNLMs to the noisy images, the CNR improved by 6.53 to 16.34 times, COV improved by 6.55 to 18.28 times, and the NNPS improved by 10^−2^ mm^2^ on average. In conclusion, our approach shows significant potential in enhancing CT image quality with anthropomorphic phantoms, thus addressing the noise issue and improving diagnostic accuracy.

## 1. Introduction

In the field of medical imaging, computed tomography (CT) using X-rays has provided significant advantages as a non-invasive examination technique, offering patient information without the need for surgery [1,2]. However, despite the various advantages of CT in providing patient information for diagnosis and patient management, there is a potential risk of radiation exposure [3]. Low-dose CT has been introduced and implemented to reduce the radiation dose in patients. However, in the process of image acquisition, noise is inevitably generated, owing to hardware errors, patient-source-related errors, and electrical interference, which degrade the diagnostic accuracy of the acquired image [4,5,6,7,8]. Particularly, low-dose CT scans exacerbate the noise issue, owing to an insufficient number of X-ray photons and increased influence of scattered rays [9,10,11,12,13].

To solve this issue, researchers have developed various noise reduction algorithms, such as the Gaussian filter, Wiener filter, and total-variation (TV) noise reduction algorithm, which are implemented using software-based image processing techniques [14,15]. However, these algorithms have the disadvantage of destroying image details during the noise reduction process. Additionally, detailed features and high-frequency signals can be reduced in the filtered region, thereby reducing the image resolution and sharpness. These limitations are due to the algorithms reducing noise without considering the similarity to neighboring pixels [16,17]. By contrast, the fast non-local means (FNLM) algorithm is a noise reduction algorithm that improves the speed of the non-local means (NLM) algorithm and uses a non-local means technique to reduce noise by considering the similarity to neighboring pixels [18,19]. This enables the accurate preservation of the image details, thereby solving the issue of image degradation. Moreover, the FNLM algorithm employs an NLM technique to explore similar patterns across the entire image and shows a higher adaptation to diverse noise types.

In addition to noise and CT image processing, several other factors contribute to the degradation of image quality, including artifacts, and blurring effects arising from device deterioration or patient motion [20,21]. To address these issues, the application of specific quality control (QC) measures is essential [22,23,24,25]. In this case, QC includes optimal device setting estimation and image acquisition processes to ensure image accuracy. Particularly, the image accuracy was maintained through quantitative evaluations based on a device-specific human phantom [26]. These phantoms serve as standardized reference objects, allowing researchers to assess image quality, identify artifacts, and optimize device settings to enhance diagnostic accuracy [27,28].

Because 3D printing techniques are cost-effective and allow for flexible design changes, large numbers of anthropomorphic phantoms can be printed for a variety of environments and purposes. In addition, 3D printing technology has shown an exponential growth, and its applications in the medical field are increasing. Three-dimensional printing techniques offer efficient production of objects featuring intricate internal structures that are appropriate for CT and magnetic resonance imaging (MRI) applications [29,30]. More recently, patient-specific surgical phantoms have been fabricated based on 3D printing technology to improve surgical planning and serve as visual aids to explain surgery to patients, assisting patients with surgical decisions. Moreover, the number of deaths caused by cardiovascular diseases is increasing rapidly in recent years, thus personalized 3D-printing phantoms are used to plan surgeries for patients with various types of cardiovascular diseases and to train clinicians to reduce procedure-related complications and improve patient care [31,32]. Based on these advantages, 3D printing techniques have been applied in various medical processing studies, such as the evaluation of reconstruction algorithms by Solomon et al. [33]. Therefore, the purpose of this study was to optimize the smoothing factor of the FNLM noise-reduction algorithm using CT images of a 3D-printed phantom. For this purpose, a phantom capable of simulating bone and brain tissues was fabricated using 3D printing to analyze the optimized smoothing factor of the FNLM noise reduction algorithm in specific anatomical structures. To quantitatively evaluate the performance of the FNLM noise reduction algorithm in CT images, local filtering techniques, such as the Gaussian filter, Wiener filter, and TV noise reduction algorithm, were compared with various noise intensity evaluation factors.

## 2. Materials and Methods

### 2.1. FNLM Noise Reduction Algorithm Modeling

The NLM noise reduction algorithm has demonstrated remarkable efficacy in noise reduction [34,35]. Moreover, NLM effectively resolves issues commonly generated by local filtering techniques for noise reduction, such as signal distortion and blurring. This is because the NLM noise reduction algorithm compares the overall geometric composition using the Euclidean distance as a weight, as opposed to using a sliding technique on the entire image, as in the Gaussian and Wiener filters. The NLM noise reduction algorithm is defined as follows [36]:

(1) $$\mathrm{NL}\left[I\right]\left(m\right)=\sum_{N\in I}\omega \left(m,n\right)I\left(n\right)$$

where the weight $\omega \left(m,n\right)$ is defined as

(2) $$\omega \left(m,n\right)=\frac{1}{Z\left(m\right)}\sum e^{-\frac{G_{\sigma }\left(\tau \right)||I\left(M+\tau \right)-I{\left(n+\tau \right)||}_{2}^{2}}{d^{2}}}$$

where τ is the number of pixels; $G_{\sigma }\left(\tau \right)$ is the Gaussian distribution with variance $\sigma^{2}$ of the number of background pixels; $||I\left(M+\tau \right)-I{\left(n+\tau \right)||}_{2}^{2}$ is the intensity difference between adjacent pixels based on the Euclidean distance value; and $Z\left(m\right)$ is the leveling constant, defined as

(3) $$Z=\sum_{n}e^{-\frac{G_{\sigma }\left(\tau \right)||I\left(M+\tau \right)-I{\left(n+\tau \right)||}_{2}^{2}}{d^{2}}}$$

The FNLM algorithm modifies the calculation of $\omega \left(m,n\right)$ from two dimensions to one dimension. The modified $\omega \left(m,n\right)$ is defined as

(4) $$\omega \left(m,n\right)=\frac{1}{Z\left(m\right)}H_{i}\left(I\left(m+s\right)-I\left(m-s\right)\right)$$

where τ is defined as n−m; s is defined as m+τ; and $H_{i}$ is defined as

(5) $$H_{i}\left(s\right)=\sum_{q=0}^{s}e^{-\frac{||I\left(q\right)-I{\left(q+\tau \right)||}_{2}^{2}}{d^{2}}}$$

The FNLM noise reduction algorithm improves processing speed approximately 30 times in comparison with the NLM noise reduction algorithm [18]. This enhancement is achieved by simplifying the process using one-dimensional computations.

### 2.2. CT Image Parameters

We used a 128-slice CT system (SOMATOM Definition AS+, Siemens Healthcare, Germany) to obtain the phantom CT images. The images of the self-produced phantom were acquired with the following parameters: 250 mAs, 120 kVp, a pitch of 0.55, a matrix size of 512 × 512, and a slice thickness of 5.0 mm. The acquired phantom images were processed using a standard algorithm.

### 2.3. 3D Printing Technology and CT Phantom Image Acquisition

**Figure 1:**
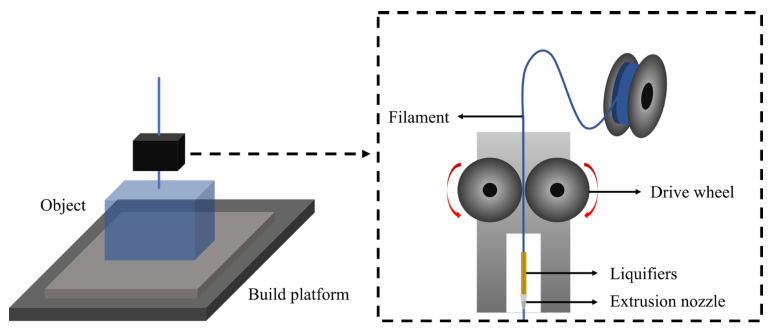
Components of the 3D printer based on the fused filament fabrication technique.

We used a 3D printer with a fused filament fabrication (FFF) technique (Ultimaker3 Extended, Ultimaker, Utrecht, the Netherlands) to produce a self-produced phantom that could imitate the human skull and brain tissues. The self-produced phantom case and blocks were produced using diverse filaments through the FFF technique of the 3D printer. Figure 1 shows the components of the 3D printer with the FFF technique.

**Figure 2:**
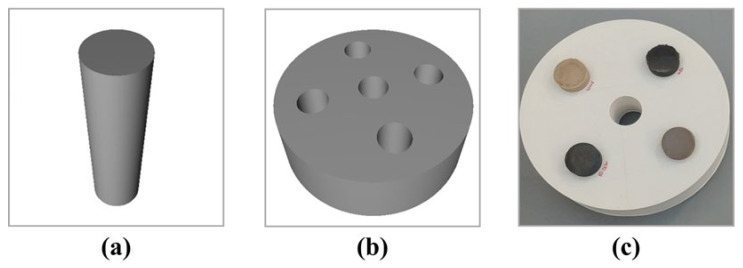
Image for manufacturing of (**a**) a blueprint of a block, (**b**) blueprint of a case, and (**c**) printed self-produced phantom.

Using SOLIDWORKS software (ver. 2019; Dassault Systèmes, Waltham, MA, USA), we designed a self-produced phantom case with dimensions of 16 cm, closely resembling the average diameter of a human skull. The case interior was perforated with five insertion holes for the blocks. Converting this design data into the STL format ensured compatibility with the 3D printer. Subsequently, CURA software (Ultimaker, the Netherlands) was employed to generate the necessary G-code and configure the hardware and software settings of the 3D printer. The self-produced phantom was then fabricated using Polylactic Acid (PLA) filaments with a density similar to that of human tissue, following the G-code instructions. Figure 2 shows the blueprint and 3D printer output for the phantom case.

Cylindrical blocks (6 and 2.5 cm in height and diameter, respectively) were designed to fit into the self-produced phantom case. These blocks were engineered to mimic the density of the human brain tissue (Table 1). For printing, we used acrylonitrile butadiene styrene (ABS), wood, and bronze filaments, including XT-CF20, a co-polyester-based carbon fiber composite material. We produced phantom blocks using a 3D printer, employing the same printing process as that used for the case. Figure 2 shows the blueprint and 3D printer outputs of the phantom blocks.

**Figure 3:**
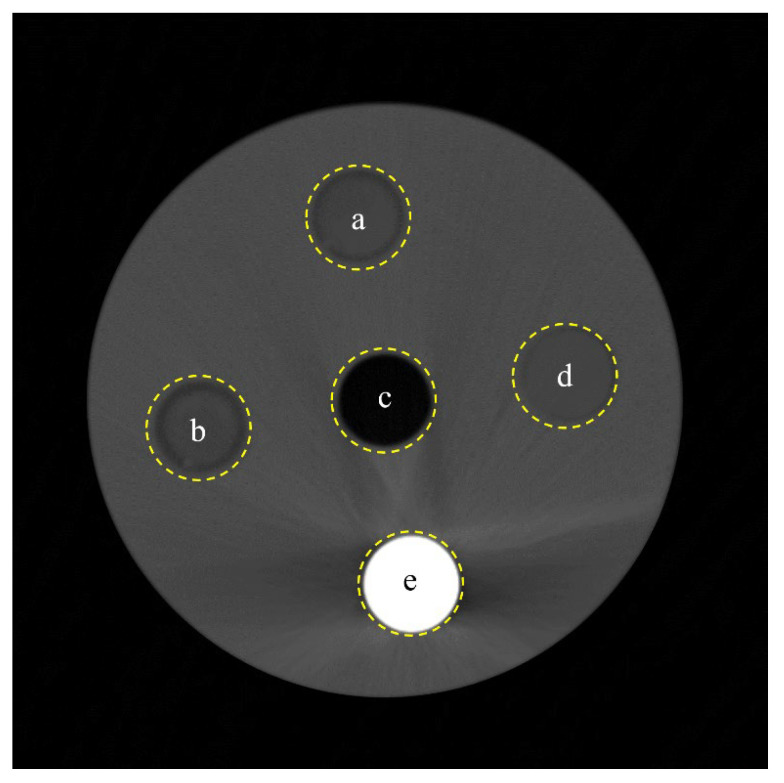
Computed tomography (CT) image of self-produced phantom with (**a**) XT-CF20, (**b**) wood, (**c**) air, (**d**) acrylonitrile butadiene styrene, and (**e**) bronze.

We obtained the self-produced phantom CT images to assess the linear attenuation coefficient. As shown in Figure 3, the inserted materials were XT-CF20, wood, air, ABS, and bronze. To simulate CT images under low-dose conditions, noisy images were obtained by adding zero-centered Gaussian noise with intensities of 0.001, 0.005, 0.01, 0.05, and 0.1 using MATLAB software (ver. 2023a; MathWorks, Boston, MA, USA). Additionally, we performed a quantitative evaluation and analysis after applying the Gaussian filter, Wiener filter, and TV noise reduction algorithm to the acquired noisy images.

### 2.4. Image Quality Evaluations

To optimize the smoothing factor of the FNLM noise reduction algorithm, we enlarged the region containing cerebrospinal fluid (CSF)-mimicking and tissue-mimicking material for visual evaluation. In this experiment, we used bronze to simulate bone; however, the CT number of bone was measured to be high because of its high density, and the signal fluctuation rate of the relative tissue was not accurately identified.

**Figure 4:**
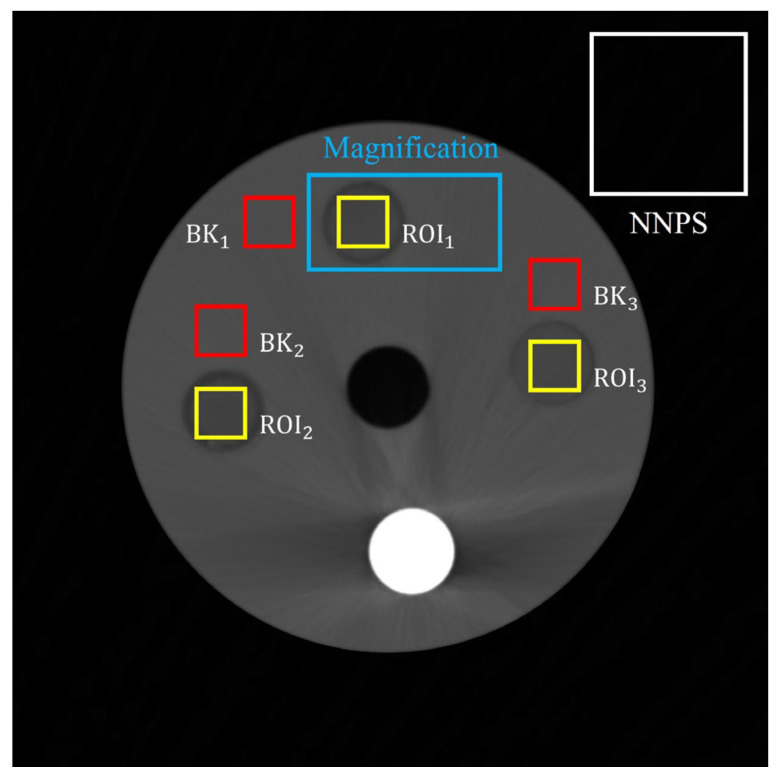
Region of interests (ROIs) setup and backgrounds for quantitative and visual evaluation.

To quantitatively evaluate the image quality, we calculated the contrast-to-noise ratio (CNR), coefficient of variation (COV), and normalized noise power spectrum (NNPS) [37,38,39,40]. We used MATLAB software (ver. 2023a) to calculate the quantitative evaluation factors. Figure 4 shows the region of interests (ROIs), in which the yellow and red boxes are the materials and background regions for noise level evaluation, the blue box is the region for visual evaluation, and the white box indicates the region for NNPS evaluation.

The CNR indicates the ratio of contrast and noise in an ROI and the background region of the same image.

(6) $$\mathrm{CNR}=\frac{\left|S_{R}-S_{\mathrm{BK}}\right|}{\sqrt{\sigma_{R}^{2}+\sigma_{\mathrm{BK}}^{2}}}$$

where $S_{R}$ and $S_{\mathrm{BK}}$ represent the average values of signal intensity in an ROI and background; $\sigma_{R}$ and $\sigma_{\mathrm{BK}}$ are standard deviations in an ROI and background, respectively. The COV was calculated to compare the degrees of deviation in the obtained phantom images.

(7) $$\mathrm{COV}=\frac{\sigma_{R}}{\mu }$$

where $\sigma_{R}$ is the standard deviation of signal intensity and μ is the average value in the ROI.

The NNPS parameter analyzes the noise variation in an image over the spatial frequency range of interest. To calculate NNPS, we first converted the noise image to the frequency domain, normalizing the power spectrum to the mean signal, and then averaging the results. The NNPS calculation is based on air.

(8) $$\mathrm{NPS}=\underset{N_{x},N_{y}\to \infty }{\lim}\left(N_{x}N_{y}\Delta x\Delta y\right)<{\left|{\mathrm{FT}}_{\mathrm{nk}}I\left(x,y\right)-S\left(x,y\right)\right|}^{2}\;=\underset{N_{x},N_{y}\to \infty }{\lim}\underset{M\to \infty }{\lim}\frac{\left(N_{x}N_{y}\Delta x\Delta y\right)}{M}\overset{M}{\underset{m=1}{\sum }}{\left|{\mathrm{FT}}_{\mathrm{nk}}I\left(x,y\right)-S\left(x,y\right)\right|}^{2}\;=\underset{N_{x},N_{y},M\to \infty }{\lim}\frac{\Delta x\Delta y}{M\cdot N_{x}N_{y}}\overset{M}{\underset{m=1}{\sum }}\langle {\left|\overset{N_{x}}{\underset{i=1}{\sum }}\overset{N_{y}}{\underset{j=1}{\sum }}\left(I\left(x_{i},y_{j}\right)-S\left(x_{i},y_{j}\right)\right)e^{-2\pi i\left(u_{n}x_{i}+v_{k}y_{i}\right)}\right|}^{2}\rangle \;{\mathrm{NNPS}}_{\mathrm{normalized}}\left(u,v\right)=\frac{\mathrm{NPS}\left(u,v\right)}{{\left(\mathrm{large}\;\mathrm{area}\;\mathrm{signal}\right)}^{2}}$$

where $I\left(x,y\right)$ denotes the average image intensity; $S\left(x,y\right)$ the average background intensity; $N_{x}$, $N_{y}$ the pixel numbers along the X- and Y-axes; and Δx, Δy the pixel sizes along the X- and Y-axes, respectively.

## 3. Results

### 3.1. Optimization of the FNLM Noise Reduction Algorithm

**Figure 5:**
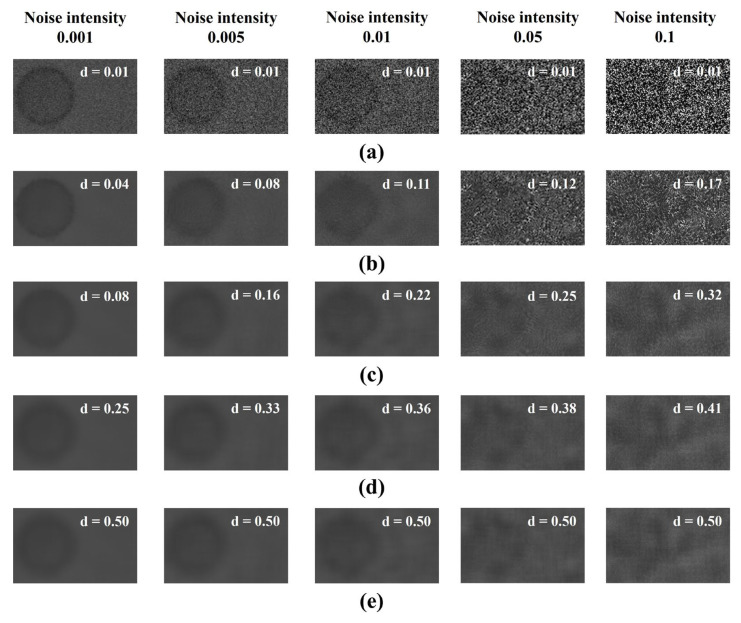
The results are magnified regions of Figure 4, using the fast non-local means (FNLM) noise reduction algorithm with various smoothing factors (*d*) on a computed tomography (CT) image with Gaussian noise added with a standard deviation: (**a**) low smoothing factor, (**b**) semi-low smoothing factor, (**c**) optimized smoothing factor, (**d**) semi-high smoothing factor, and (**e**) high smoothing factor.

We performed a visual evaluation to derive the optimal value of the smoothing factor, which is a parameter of the FNLM noise reduction algorithm. Figure 5 shows the results of the FNLM noise reduction algorithm with various smoothing factors applied to a CT image with added Gaussian noise, with standard deviations of 0.001, 0.005, 0.01, 0.05, and 0.1. In Figure 5, the reason for choosing a low smoothing factor (*d* = 0.01) and a high smoothing factor (*d* = 0.50) was to emphasize that a low smoothing factor is insufficient for noise reduction, and that a high smoothing factor effectively reduces noise; however, it results in significant information loss. From the visual evaluation, we observed that the lower the smoothing factor, the more noise remained, and the higher the smoothing factor, the more effectively the noise was reduced. However, as the smoothing factor increased, we observed a loss of edge signal, a blurring effect, and a decrease in contrast between the two materials. At a smoothing factor of 0.50, as shown in Figure 5, we observed that the noise was effectively reduced; however, blurring effects were generated at low noise intensities, and the grid artifacts were enhanced at high noise intensities.

**Figure 6:**
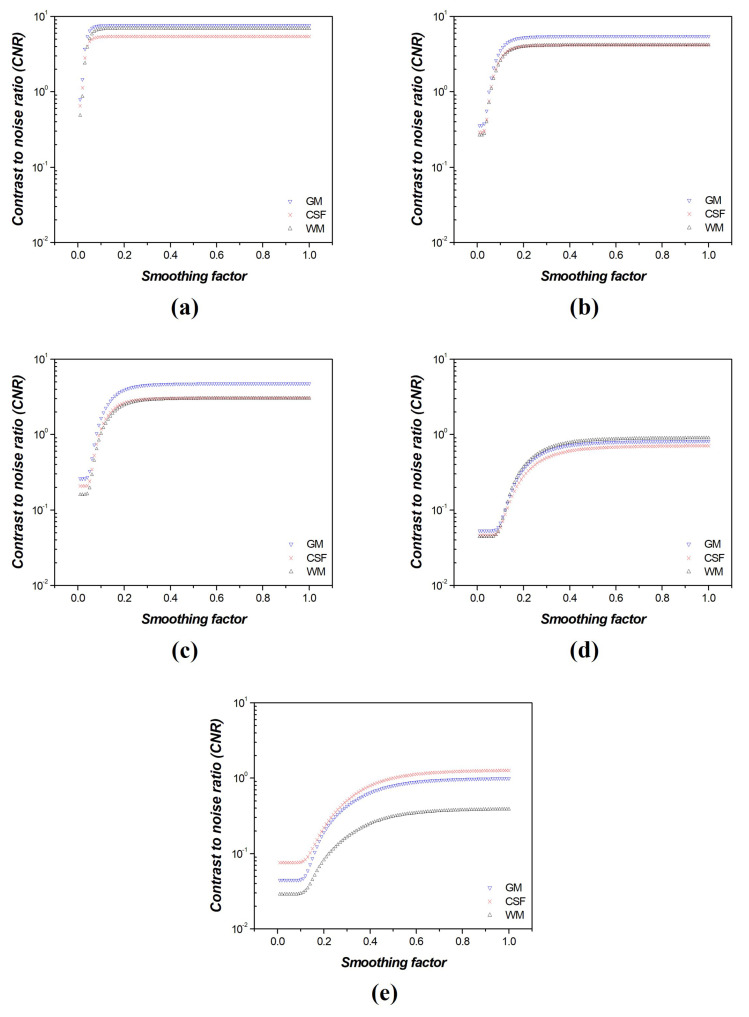
Contrast−to−noise ratio (CNR) results according to region of interests (ROIs) of Figure 4 in computed tomography (CT) images of the self−produced phantom with various noise intensities: (**a**) 0.001, (**b**) 0.005, (**c**) 0.01, (**d**) 0.05, and (**e**) 0.1.

To analyze the performance of the FNLM noise reduction algorithm and optimize the smoothing factor for each noise intensity, we calculated CNR and COV as quantitative evaluation factors. The quantitative evaluation results confirmed that the CSF, gray matter (GM), and white matter (WM) regions showed improved image quality. Figure 6 shows the CNR results in the measured CSF, GM, and WM regions for images with a smoothing factor for which the FNLM noise reduction algorithm increased from 0.01 to 1.00 at various noise intensities.

An analysis of the CNR graph revealed a significant increase in the CNR for the CSF, GM, and WM regions, followed by a decrease in the slope after reaching a certain point, converging to a consistent value. Additionally, we confirmed that as the noise intensity increased, the overall CNR value decreased, and the smoothing factor value of the FNLM noise reduction algorithm increased in the region where the slope became constant.

**Figure 7:**
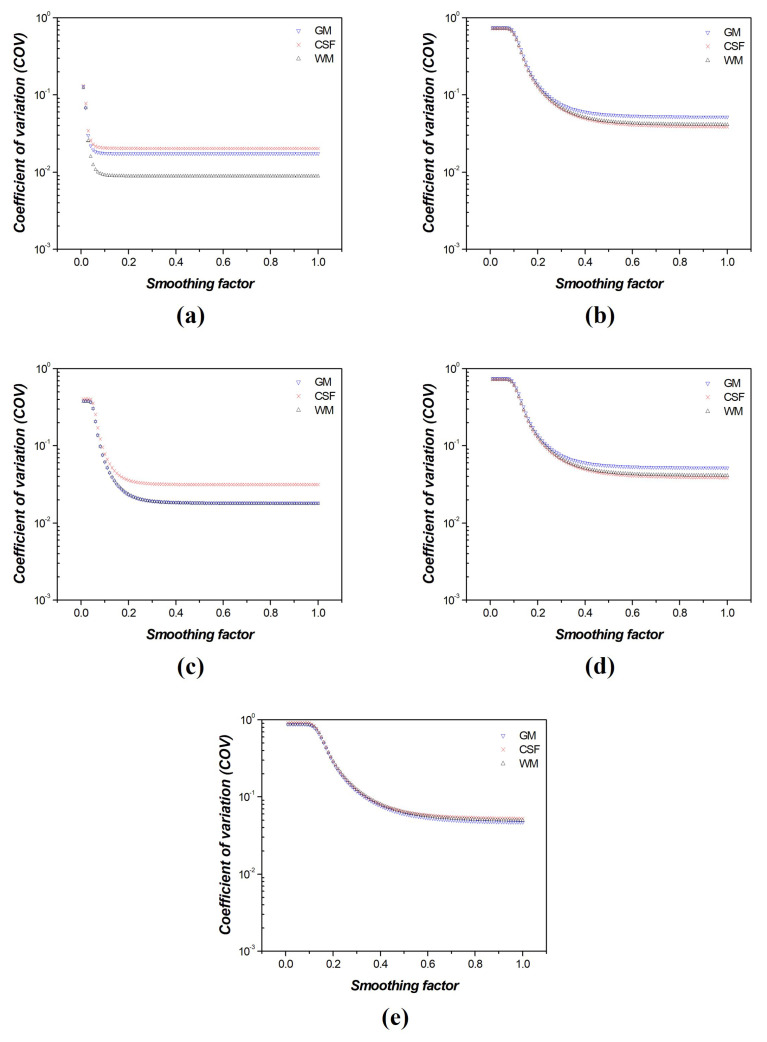
Coefficient of variation (COV) results according to region of interests (ROIs) of Figure 4 in computed tomography (CT) images of the self−produced phantom with various noise intensities: (**a**) 0.001, (**b**) 0.005, (**c**) 0.01, (**d**) 0.05, and (**e**) 0.1.

Figure 7 shows the COV results for the measured CSF, GM, and WM regions, where the smoothing factor for the FNLM noise reduction algorithm increased from 0.01 to 1.00 at various noise intensities. By analyzing the COV graph, we observed that the CSF, GM, and WM graphs showed a rapid decrease in slope and then converged to a consistent value. Moreover, as the noise intensity increased, the average COV value increased, and the smoothing factor of the FNLM noise reduction algorithm increased in areas where the slope was constant. When calculating the slope of the CNR graphs, we found that the slope decreased by half when the smoothing factors of the FNLM noise reduction algorithm were set to 0.12, 0.24, 0.33, 0.30, and 0.40, at the noise intensities of 0.001, 0.005, 0.01, 0.05, and 0.1, respectively. Moreover, when calculating the slope of the COV graphs, we confirmed that the slope decreased by half when the smoothing factors of the FNLM noise reduction algorithm were 0.04, 0.07, 0.10, 0.19, and 0.24, at the noise intensities of 0.001, 0.005, 0.01, 0.05, and 0.1, respectively. Therefore, the average values of the smoothing factor, specifically 0.08, 0.16, 0.22, 0.25, and 0.32, at the point where the slopes of the CNR and COV were halved, were derived as the optimized values at the noise intensities of 0.001, 0.005, 0.01, 0.05, and 0.1, respectively.

Thus, the optimized smoothing factor value was determined based on the change in the CNR and COV values (slope = 0.01) for CT images with various smoothing factors and noise intensities of 0.001, 0.005, 0.01, 0.05, and 0.1, respectively. In addition, the average values of the CNR and COV were used to set the optimized values of the smoothing factor, ensuring a balance between noise reduction and resolution enhancement.

### 3.2. Comparative Evaluation of the FNLM Algorithm and Conventional Noise Reduction Methods

To evaluate the efficacy of the optimized FNLM, we conducted a comparative evaluation with local filtering techniques for noise reduction, such as the Gaussian filter, Wiener filter, and the TV noise reduction algorithm.

**Figure 8:**
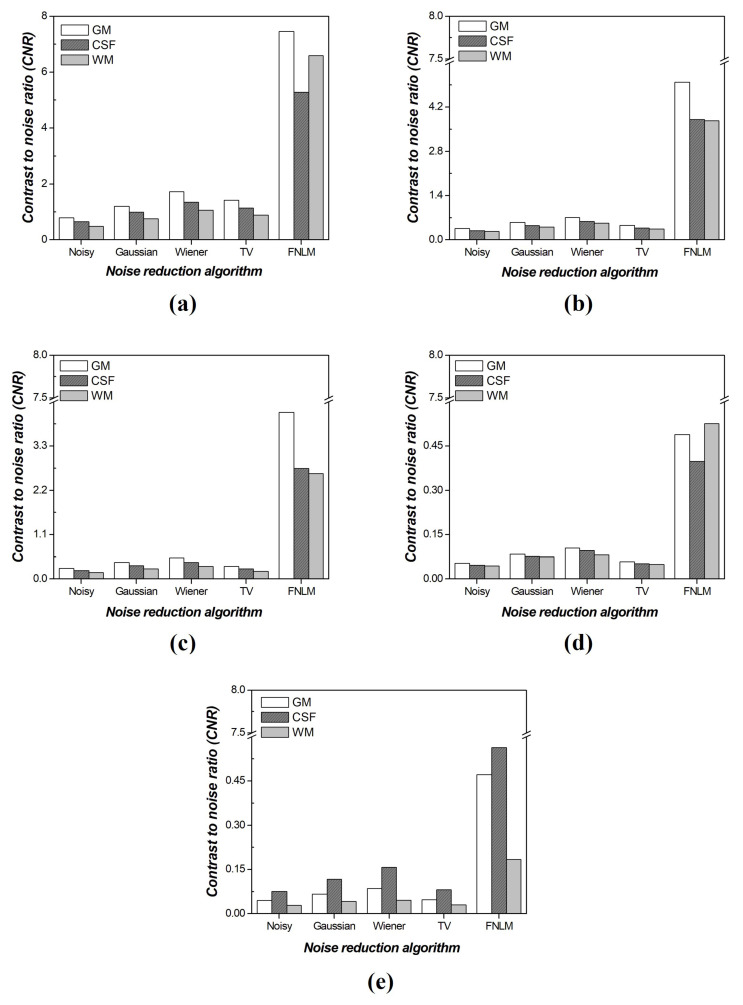
Comparative contrast-to-noise ratio (CNR) evaluation results of optimized fast non-local means (FNLM), local filtering techniques, and total-variation (TV) noise reduction algorithm on self-produced phantom with various noise intensities: (**a**) 0.001, (**b**) 0.005, (**c**) 0.01, (**d**) 0.05, and (**e**) 0.1.

Figure 8 shows the CNR results of the optimized FNLM algorithm, local filtering techniques, and the TV noise reduction algorithm for each material. According to the analysis results, the optimized FNLM exhibited superior values across various noise intensities. At a noise intensity of 0.001, the performance order was FNLM, Wiener filter, TV algorithm, Gaussian filter, and noisy image. For all other noise intensities except 0.001, improved results were observed in the order of FNLM, Wiener filter, Gaussian filter, TV algorithm, and noisy image. Particularly, when comparing the noisy image with the optimized FNLM, we confirmed that the CNR value improved by an average factor of about 10.33 times at a noise intensity of 0.001. Additionally, the CNR value improved by approximately 13.77 and 15.13 times at the noise intensities of 0.005 and 0.01, respectively. Finally, we confirmed that the CNR value improved by approximately 9.92 and 8.19 times at the noise intensities of 0.05 and 0.1, respectively (Table 2).

**Figure 9:**
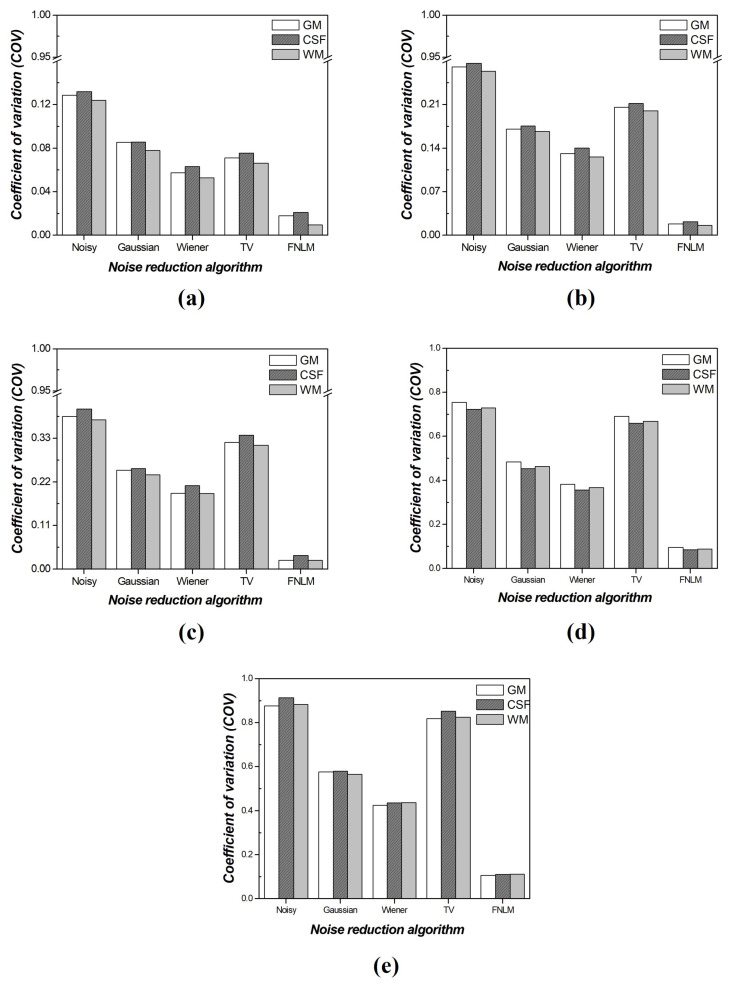
Comparative coefficient of variation (COV) evaluation results of optimized fast non-local means (FNLMs), local filtering techniques, and total-variation (TV) noise reduction algorithm on self-produced phantom with various noise intensities: (**a**) 0.001, (**b**) 0.005, (**c**) 0.01, (**d**) 0.05, and (**e**) 0.1.

Figure 9 presents the COV results of the optimized FNLM algorithm, local filtering techniques, and the TV noise reduction algorithm for each material. The optimized FNLM exhibited superior values across various noise intensities. At a noise intensity of 0.001, the performance order was FNLM, Wiener filter, TV algorithm, Gaussian filter, and noisy image. For all other noise intensities except 0.001, enhanced results were observed in the order of FNLM, Wiener filter, Gaussian filter, TV algorithm, and noisy image. When comparing the noisy image with the optimized FNLM, we confirmed that, on average, the COV value improved by approximately 9.92 and 15.22 times at the noise intensities of 0.001 and 0.005, respectively. Furthermore, at the noise intensities of 0.01 and 0.05, we confirmed enhancements of about 16.01 and 8.26 times, respectively. Moreover, we observed improvements of approximately 8.16 times at a noise intensity of 0.1 (Table 3).

**Figure 10:**
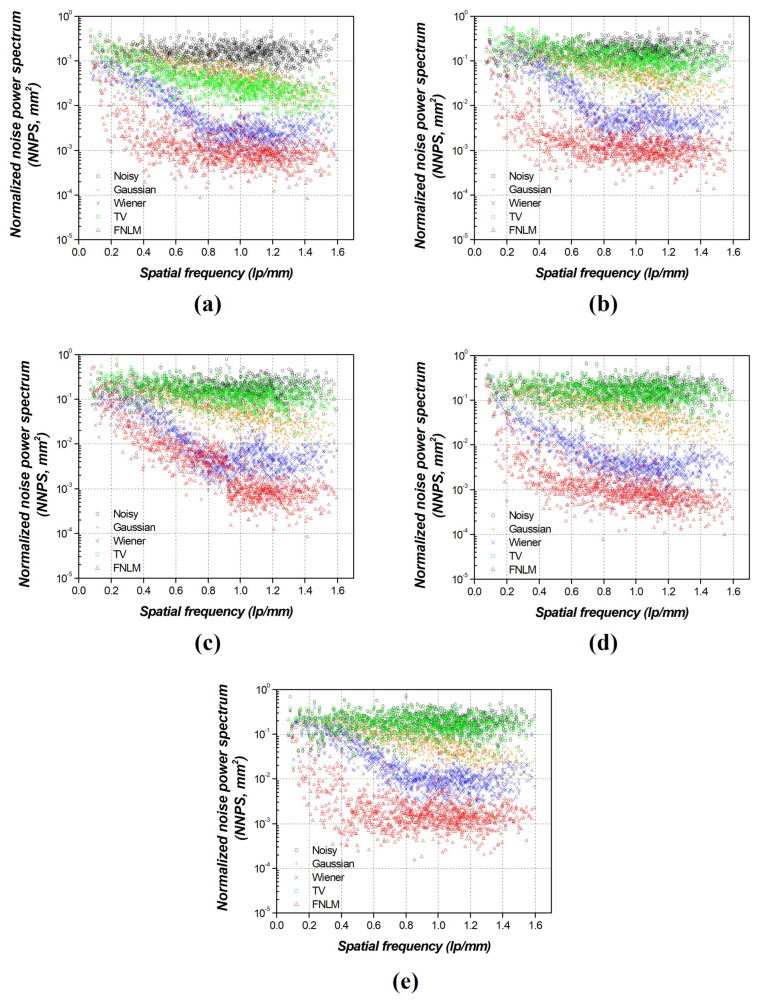
Comparative normalized noise power spectrum evaluation results of optimized fast non-local means (FNLMs), local filtering techniques, and total-variation (TV) noise reduction algorithm on self-produced phantom with various noise intensities: (**a**) 0.001, (**b**) 0.005, (**c**) 0.01, (**d**) 0.05, and (**e**) 0.1.

Figure 10 shows the results of the NNPS after applying the local filtering techniques, the TV noise reduction algorithm, and the optimized FNLM noise reduction algorithm. In all the images, with various noise intensities, the NNPS value gradually decreased with an increase in the spatial frequency. When we used the optimized FNLM algorithm, the gradual decrease in the NNPS was approximately 10^−2^ mm^2^ compared to that of the noisy image, with an increase in the spatial frequency at the noise intensities of 0.001, 0.005, 0.01, 0.05, and 0.1.

## 4. Discussion

In the field of radiology, X-ray-based CT systems play a critical role in non-invasive medical imaging, facilitating patient diagnosis [41]. However, these systems have challenges such as device quality deterioration, detector performance degradation, and potential patient motion during CT scans, which can lead to diagnostic errors [8]. To address these issues, various techniques have been proposed in numerous studies, particularly those focusing on software-based algorithms for CT image processing. However, local filtering techniques and TV noise reduction algorithms are limited in terms of image quality preservation and efficiency [42]. To overcome these limitations, an NLM noise reduction algorithm was developed. The NLM noise reduction algorithm achieves interior pixel value equalization while preserving edges by utilizing ROIs and weight calculations based on adjacent pixel intensities and Euclidean distances [4,43]. However, the NLM noise reduction algorithm has time constraints because of its computational complexity. To address this computational challenge, we used the FNLM noise reduction algorithm, which reduces computational complexity by transforming the weight calculations from two dimensions to one dimension [22].

In the field of medical imaging, numerous studies have focused on the application of 3D printing technology [44,45,46,47,48,49]. Particularly, these studies focused on tools for implantation or invasive procedures in the human body, phantoms for planning surgical operations, and image acquisition precision based on 3D medical images [50,51,52]. Despite this progress, research on the printing of phantoms to evaluate image quality has received limited attention. Thus, in this study, we developed a self-produced phantom to evaluate image quality and examined its usefulness in the image quality assessment of medical images.

To assess the effectiveness of the FNLM noise reduction algorithm, a comparative evaluation was conducted using local filtering techniques and a TV noise reduction algorithm. Additionally, a self-produced phantom was fabricated using 3D printing technology. The evaluation focused on noise parameters, including CNR, COV, and NNPS. These parameters are used as critical quantitative metrics for evaluating the efficacy of the noise reduction algorithms.

As a result of the visual comparative evaluation in Figure 5, we found that noise remained when low smoothing factor values were applied. Moreover, we observed that as the smoothing factor increased significantly, noise was effectively reduced; however, blurring artifacts were generated at low noise intensities and grid artifacts were intensified at high noise intensities. Furthermore, the contrast between the CSF and the tissue decreased as the smoothing factor increased because, as the smoothing factor increases, the interference between the signals of the two tissues increases due to excessive smoothing from the increased distance weight of the FNLM algorithm. Consequently, the signals of other tissues are invaded, resulting in the loss of high-frequency information in some areas, and the difference in signal values between the CSF and tissue parts becomes similar.

The CNR and COV graphs indicated a higher contrast improvement and noise reduction efficiency as the slope changes rapidly. Based on these tendencies, we identified regions where the contrast was reduced by blending the signals from the two different tissues using the CNR measurement results. In addition, the COV results showed regions where the blurring effect had a greater impact on the image than noise reduction. To derive the optimized smoothing factor for CNR and COV, we considered the region where the slope decreased by more than half (where the improvement rate dropped sharply). Subsequently, to balance noise reduction and contrast, the optimization of the FNLM noise reduction algorithm was set to the mean value of the optimized smoothing factor values measured in the CNR and COV. In addition, we observed that the degree of improvement in image quality decreased as the noise intensity increased, and we identified an increase in the value of the smoothing factor at points with a consistent slope. Consequently, the optimized smoothing factors for the FNLM noise reduction algorithm were determined to be 0.08, 0.16, 0.22, 0.25, and 0.32 for the CT images with added Gaussian noise intensities of 0.001, 0.005, 0.01, 0.05, and 0.1, respectively.

Based on these results, we derived the optimized values for the smoothing factor of the FNLM algorithm and conducted a quantitative comparative evaluation of the images using the optimized smoothing factor, local filtering techniques, and the TV noise reduction algorithm. After applying the optimized FNLM noise reduction algorithm at a noise intensity of 0.005, the CNR values in various ROIs improved by 6.55 to 10.97 times, and the COV values improved by 6.66 to 13.33 times. Moreover, at a noise intensity of 0.01, the CNR values improved by approximately 6.85 to 13.80 times, and the COV values improved by 6.20 to 15.19 times. At a noise intensity of 0.1, the CNR values improved by 3.58 to 10.02 times, and the COV values improved by 3.92 to 7.74 times. In addition, the NNPS of the image subjected to the optimized FNLM noise reduction algorithm improved by approximately $10^{-2}\;{\mathrm{mm}}^{2}$ compared to that of the noisy image at the noise intensities of 0.001, 0.005, 0.01, 0.05, and 0.1. Numerous studies have been conducted on the FNLM noise reduction algorithm, with a primary focus on improving image quality. Therefore, our results indicate that the FNLM noise reduction algorithm is effective in reducing noise in CT images and outperforms local noise reduction algorithms under low-dose settings with high noise levels. Additionally, the FNLM noise reduction algorithm effectively enhanced the CNR and COV in the CSF, GM, and WM regions, regardless of the noise intensity.

In our study, we fabricated a phantom with the approximate dimensions of a human head using a 3D printer by employing filaments composed of various materials to simulate human tissues. Subsequently, we quantitatively evaluated the FNLM noise reduction algorithm using the CNR, COV, and NNPS parameters, which reflect both noise and image quality of a self-produced phantom image. A limitation of this study is that the bone region was not evaluated; a self-produced phantom block was produced using bronze to mimic bone material, but the signal was too strong to measure. In addition, the bronze block caused metallic artifacts because of its high density, which may have affected the performance of the local filtering techniques, TV noise reduction algorithm, and FNLM noise reduction algorithm. To demonstrate the performance of the FNLM denoising algorithm, a quantitative evaluation was conducted by setting up ROIs to minimize the effects of metallic artifacts. Although the results of this study demonstrate that the FNLM algorithm has useful denoising efficiency, we plan to fabricate other blocks that can better simulate bone material and to conduct a phantom study that includes bone parts in the future to achieve more accurate results.

Our study demonstrated the effectiveness of the FNLM noise reduction algorithm for CT noise reduction and significant image quality enhancement. In the clinical field, low-dose CT has been used to address the issue of radiation exposure. However, low-dose CT scans inevitably introduce noise because of an insufficient number of photons during image acquisition [53,54]. Based on the results of our study, we anticipate that the FNLM algorithm can effectively reduce the noise generated in low-dose CT images, thereby significantly reducing radiation exposure.

Recently, an adaptive non-local means (ANLM) algorithm has been proposed, which improves on the traditional NLM by dynamically adjusting parameters based on noise characteristics [55]. Compared to the traditional NLM algorithm, which requires the manual optimization of the smoothing factor, the ANLM algorithm automatically detects the noise characteristics and dynamically adjusts parameters such as the smoothing factor and search window size. This allows the ANLM algorithm to automatically derive optimized values and perform more flexibly and effectively under various noise conditions. Although previous studies have demonstrated the efficiency of the ANLM algorithm, there is limited research comparing the performance of the ANLM algorithm and the FNLM algorithm. Therefore, a comparative performance study between the optimized smoothing factor of the FNLM algorithm and the automatic optimization technique of the ANLM is needed to identify the advantages and disadvantages of each in various noise conditions.

Additionally, this study suggests the possibility of improving medical imaging processes using 3D printing, leading to higher diagnostic accuracy. Traditional 3D printing techniques have been used in certain parts of the medical field, including procedural tools, assistive devices, and implants [56,57,58,59,60]. However, this study demonstrates the feasibility of applying 3D printer-based phantoms in the field of medical image processing. Moreover, the 3D printing technique offers significant advantages in terms of cost effectiveness and reproducibility, making it suitable for mass production [61,62,63,64]. Because of these advantages, the application of 3D printing in deep learning is also feasible [65,66]. Our study highlights several advantages of using denoised images to construct a deep learning dataset. Denoised images offer improved quality, enhancing the accuracy of deep learning model training and the generalization capability of these models [67,68,69]. Using 3D-printing phantoms to build datasets for deep learning can minimize ethical concerns compared to using patient data. Specifically, using phantoms to acquire images avoids issues related to patient privacy and reduces radiation exposure, which are significant ethical considerations. Furthermore, 3D-printing phantoms provide repeatability and reproducibility, making them suitable for QC purposes. Our study results show that the integration of 3D printing and phantom fabrication techniques has the potential to create innovative technologies and algorithms to improve medical image quality. This approach addresses the ethical issues of data scarcity and radiation exposure, as well as leveraging the consistent and controllable characteristics of phantoms to generate high-quality datasets for deep learning applications. Therefore, we expect that when 3D printing technology is properly utilized in medical imaging studies, it will contribute to the improvement of medical imaging quality, as well as the advancement of deep learning technology.

## 5. Conclusions

In this study, we modeled and applied the FNLM noise reduction algorithm to reduce noise in medical images acquired from CT systems. To evaluate image quality, we fabricated a self-produced phantom using a 3D printer. In addition, we conducted a quantitative analysis by comparing the FNLM algorithm with local filtering techniques and the TV noise reduction algorithm to confirm the effectiveness of the FNLM noise reduction approach. In the phantom study, we confirmed considerable improvements across all quantitative evaluation factors with the implementation of the FNLM noise reduction algorithm, surpassing the performances of local filtering techniques and the TV noise reduction algorithm. These results suggest that the FNLM algorithm can effectively replace local filtering techniques and the TV noise reduction algorithm. Furthermore, this study demonstrates that self-produced phantoms using 3D printers can be adaptively applied in the medical image processing field.

## Figures and Tables

**Table 1:** Attenuation coefficient of the reference tissue and filament materials.

Reference Tissue	Attenuation Coefficient (cm^−1^)	Filament Materials	Attenuation Coefficient (cm^−1^)
Cerebrospinal fluid (CSF)	0.207	XT-CF20	0.208
Gray matter (GM)	0.212	Wood	0.213
White matter (WM)	0.213	ABS	0.214
Bone	0.528	Bronze	0.839

**Table 2:** Contrast-to-noise ratio results for computed tomography (CT) images with noise intensities of 0.001, 0.005, 0.01, 0.05, and 0.1, respectively.

Noise Intensity	Algorithm	CSF (XT-CT20)	GM (Wood)	WM (ABS)
0.001	Noisy	0.652	0.793	0.486
	Gaussian	0.994	1.199	0.754
	Wiener	1.351	1.727	1.062
	TV	1.138	1.416	0.881
	FNLM	5.278	7.458	6.588
0.005	Noisy	0.289	0.358	0.264
	Gaussian	0.448	0.549	0.406
	Wiener	0.581	0.710	0.531
	TV	0.376	0.462	0.343
	FNLM	3.806	4.982	3.766
0.01	Noisy	0.207	0.261	0.160
	Gaussian	0.331	0.406	0.253
	Wiener	0.406	0.523	0.312
	TV	0.251	0.316	0.193
	FNLM	2.740	4.134	2.615
0.05	Noisy	0.046	0.053	0.044
	Gaussian	0.076	0.083	0.074
	Wiener	0.096	0.104	0.081
	TV	0.051	0.058	0.049
	FNLM	0.397	0.488	0.526
0.1	Noisy	0.076	0.044	0.028
	Gaussian	0.117	0.066	0.042
	Wiener	0.157	0.085	0.045
	TV	0.081	0.047	0.030
	FNLM	0.563	0.471	0.183

**Table 3:** Coefficient of variation results for computed tomography (CT) images with noise intensities of 0.001, 0.005, 0.01, 0.05, and 0.1, respectively.

Noise Intensity	Algorithm	CSF (XT-CT20)	GM (Wood)	WM (ABS)
0.001	Noisy	0.131	0.128	0.123
	Gaussian	0.085	0.085	0.077
	Wiener	0.063	0.057	0.052
	TV	0.075	0.071	0.066
	FNLM	0.020	0.017	0.009
0.005	Noisy	0.276	0.270	0.263
	Gaussian	0.175	0.170	0.166
	Wiener	0.140	0.131	0.126
	TV	0.211	0.205	0.200
	FNLM	0.021	0.018	0.015
0.01	Noisy	0.403	0.384	0.376
	Gaussian	0.254	0.249	0.238
	Wiener	0.211	0.191	0.190
	TV	0.337	0.319	0.312
	FNLM	0.034	0.021	0.021
0.05	Noisy	0.722	0.754	0.729
	Gaussian	0.453	0.483	0.463
	Wiener	0.356	0.383	0.368
	TV	0.659	0.690	0.667
	FNLM	0.084	0.095	0.088
0.1	Noisy	0.913	0.876	0.882
	Gaussian	0.579	0.575	0.565
	Wiener	0.436	0.425	0.436
	TV	0.852	0.818	0.824
	FNLM	0.110	0.106	0.111

## Data Availability

The raw data supporting the conclusions of this article will be made available by the authors on request.

## References

[1] Brooks R.A., Di Chiro G.D. (1976). Principles of computer assisted tomography (CAT) in radiographic and radioisotopic imaging. Phys. Med. Biol..

[2] Otomo N., Funao H., Yamanouchi K., Isogai N., Ishii K. (2022). Computed Tomography-Based Navigation System in Current Spine Surgery: A narrative Review. Medicina.

[3] Phase B.V. (2006). Health Risks from Exposure to Low Levels of Ionizing Radiation.

[4] Diwakar M., Kumar P., Singh A.K. (2020). CT image denoising using NLM and its method noise thresholding. Multimed. Tool. Appl..

[5] Duan X., Wang J., Leng S., Schmidt B., Allmendinger T., Grant K., Flohr T., McCollough C.H. (2013). Electronic noise in CT detectors: Impact on image noise and artifacts. AJR Am. J. Roentgenol..

[6] Song H., Chen L., Cui Y., Li Q., Wang Q., Fan J., Yang J., Zhang L. (2022). Denoising of MR and CT images using cascaded multi-supervision convolutional neural networks with progressive training. Neurocomputing.

[7] Kijewski M.F., Judy P.F. (1987). The noise power spectrum of CT images. Phys. Med. Biol..

[8] Diwakar M., Kumar M. (2018). A review on CT image noise and tis denoising. Biomed. Signal Process Control.

[9] Veldkamp W.J.H., Kroft L.J., Geleijns J. (2009). Dose and perceived image quality in chest radiography. Eur. J. Radiol..

[10] Andria G., Attivissimo F., Di Nisio A., Lanzolla A.M., Maiorana A., Mangiatini M., Spadavecchia M. (2017). Dosimetric characterization and image quality assessment in breast tomosynthesis. IEEE Trans. Instrum. Meas..

[11] Mettler F.A., Wiest P.W., Locken J.A., Kelsey C.A. (2000). CT scanning: Patterns of use and dose. J. Radiol. Prot..

[12] Naidich D.P., Marshall C.H., Gribbin C., Arams R.S., McCauley D.I. (1990). Low-dose CT of the lungs: Preliminary observations. Radiology.

[13] Goo H.W. (2012). CT radiation dose optimization and estimation: An update for radiologists. Korean J. Radiol..

[14] Bharati S., Khan T.Z., Podder P., Hung N.Q. (2021). A comparative analysis of image denoising problem: Noise models, denoising filters and applications. Cogn. Internet Med. Things Smart Healthc. Serv. Appl..

[15] Tian Z., Jia X., Yuan K., Pan T., Jiang S.B. (2011). Low-dose CT reconstruction via edge-preserving total variation regularization. Phys. Med. Biol..

[16] Wang M., Zheng S., Li X., Qin X. A new image denoising method based on Gaussian filter. Proceedings of the 1st International Conference on Information Science, Electronics, and Electrical Engineering.

[17] Ramesh G., Logeshwaran J., Gowri J., Mathew A. (2022). The management and reduction of digital noise in video image processing by using transmission based noise elimination scheme. CTACT J. Image Video Process..

[18] Dauwe A., Goossens B., Luong H.Q., Philips W. (2008). A fast non-local image denoising algorithm. Image Process. Algorithms Syst. VI..

[19] Jang M.Y., Park C.R., Kang S.H., Lee Y. (2020). Experimental study of the fast non-local means noise reduction algorithm using the Hoffman 3D brain phantom in nuclear medicine SPECT image. Optik.

[20] Faulkner K., Moores B.M. (1984). Noise and contrast detection in computed tomography images. Phys. Med. Biol..

[21] McCollough C.H., Yu L., Kofler J.M., Leng S., Zhang Y., Li Z., Carter R.E. (2015). Degradation of CT low-contrast spatial resolution due to the use of iterative reconstruction and reduced dose levels. Radiology.

[22] Mansour Z., Mokhtar A., Sarhan A., Ahmed M.T., Ei-El-Diasty T. (2016). Quality control of CT image using American College of Radiology (ACR) phantom. Egypt. J. Rad. Nucl. Med..

[23] Pauwels R., Stamatakis H., Manousaridis G., Walker A., Michielsen K., Bosmans H., SEDENTEXCT Project Consortium (2011). Development and applicability of a quality control phantom for dental cone-beam CT. J. Appl. Clin. Med. Phys..

[24] Nowik P., Bujila R., Poludniowski G., Fransson A. (2015). Quality control of CT systems by automated monitoring of key performance indicators: A two-year study. J. Appl. Clin. Med. Phys..

[25] Pauchard Y., Liphardt A.M., Macdonald H.M., Hanley D.A., Boyd S.K. (2012). Quality control for bone quality parameters affected by subject motion in high-resolution peripheral quantitative computed tomography. Bone.

[26] Leng S., Yu L., Vrieze T., Kuhlmann J., Chen B., McCollough C.H. (2015). Construction of realistic liver phantoms from patient images using 3D printer and its application in CT image quality assessment. Proc. SPIE Int. Soc. Opt. Eng..

[27] Miller M.A., Hutchins G.D. Development of anatomically realistic PET and PET/CT phantoms with rapid prototyping technology. Proceedings of the 6th IEEE Nuclear Science Symposium Conference Record.

[28] Nute J.L.N., Jacobsen M.C., Stefan W., Wei W., Cody D.D. (2018). Development of a dual-energy computed tomography quality control program: Characterization of scanner response and definition of relevant parameters for a fast-kVp switching dual-energy computed tomography system. Med. Phys..

[29] Filippou V., Tsoumpas C. (2018). Recent advances on the development of phantoms using 3D printing for imaging with CT, MRI, PET, SPECT, and ultrasound. Med. Phys..

[30] Diekhoff T., Scheel M., Kress W., Hamm B., Jahnke P. (2021). Dual-energy computed tomography of the neck-optimizing tube current settings and radiation dose using a 3D-printed patient phantom. Quant. Imaging Med. Surg..

[31] Higgins M., Leung S., Radacsi N. (2022). 3D printing surgical phantoms and their role in the visualization of medical procedures. Ann. 3D Print. Med..

[32] Sun Z. (2020). Clinical applications of patient-specific 3D printed models in cardiovascular disease: Current status and future directions. Biomolecules.

[33] Solomon J., Samei E. (2014). Quantum noise properties of CT images with anatomical textured backgrounds across reconstruction algorithms: FBP and SAFIRE. Med. Phys..

[34] Buades A., Coll B., Morel J.-M. A non-local algorithm for image denoising. Proceedings of the 2nd IEEE Computer Society Conference on Computer Vision and Pattern Recognition (CVPR’05).

[35] Buades A., Coll B., Morel J.M. (2005). A review of image denoising algorithms, with a new one. Multiscale Model. Simul..

[36] Shim J., Yoon M., Lee M.J., Lee Y. (2021). Utility of fast non-local means (FNLM) filter for detection of pulmonary nodules in chest CT for pediatric patient. Phys. Med..

[37] Li X., Huang W., Rooney W.D. (2012). Signal-to-noise ratio, contrast-to-noise ratio and pharmacokinetic modeling considerations in dynamic contrast-enhanced magnetic resonance imaging. Magn. Reson. Imaging..

[38] Martin R. (2001). Noise power spectral density estimation based on optimal smoothing and minimum statistics. IEEE Trans. Speech Audio Process..

[39] Tian L. (2005). Inferences on the common coefficient of variation. Stat. Med..

[40] Engel K.J., Herrmann C., Zeitler G. (2008). X-ray scattering in single-and-dual source CT. Med. Phys..

[41] Liguori C., Frauenfelder G., Massaroni C., Saccomandi P., Giurazza F., Pitocco F., Marano R., Schena E. (2015). Emerging clinical applications of computed tomography. Med Devices Evid. Res..

[42] Zhu Y., Zhao M., Zhao Y., Li H., Zhang P. (2012). Noise reduction with low dose CT data based on a modified ROF model. Opt. Express.

[43] Rudin L.I., Osher S., Fatemi E. (1992). Nonlinear total variation based noise removal algorithms. Phys. D Nonlinear Phenom..

[44] Hazelaar C., van Eijnatten M., Dahele M., Wolff J., Forouzanfar T., Slotman B., Verbakel W.F. (2018). Using 3D printing techniques to create an anthropomorphic thorax phantom for medical imaging purposes. Med. Phys..

[45] Yan Q., Dong H., Su J., Han J., Song B., Wei Q., Shi Y. (2018). A review of 3D printing technology for medical applications. Engineering.

[46] Tack P., Victor J., Gemmel P., Annemans L. (2016). 3D-printing techniques in a medical setting: A systematic literature review. Biomed. Eng. OnLine.

[47] Ventola C.L. (2014). Medical applications for 3D printing: Current and projected uses. Pharm. Ther..

[48] Durfee W.K., Iaizzo P.A. (2019). Medical applications of 3D printing. Engineering in Medicine.

[49] Liaw C.Y., Guvendiren M. (2017). Current and emerging applications of 3D printing in medicine. Biofabrication.

[50] Lin H.H., Lonic D., Lo L.J. (2018). 3D printing in orthognathic surgery-A literature review. J. Formos. Med. Assoc..

[51] Qiu K., Haghiashtiani G., McAlpine M.C. (2018). 3D printed organ models for surgical applications. Annu. Rev. Anal. Chem..

[52] Giannopoulos A.A., Steigner M.L., George E., Barile M., Hunsaker A.R., Rybicki F.J., Mitsouras D. (2016). Cardiothoracic applications of 3D printing. J. Thorac. Imaging.

[53] Wolterink J.M., Leiner T., Viergever M.A., Išgum I. (2017). Generative adversarial networks for noise reduction in low-dose CT. IEEE Trans. Med. Imaging.

[54] Ohno Y., Takenaka D., Kanda T., Yoshikawa T., Matsumoto S., Sugihara N., Sugimura K. (2012). Adaptive iterative dose reduction using 3D processing for reduced- and low-dose pulmonary CT: Comparison with standard-dose CT for image noise reduction and radiological findings. AJR Am. J. Roentgenol..

[55] Verma R., Pandey R. Non local means algorithm with adaptive isotropic search window size for image denoising. Proceedings of the Annual IEEE India Conference (INDICON).

[56] Wang Z., Yang Y. (2021). Application of 3D printing in implantable medical devices. BioMed Res. Int..

[57] Michalski M.H., Ross J.S. (2014). The shape of things to come: 3D printing in medicine. JAMA.

[58] Abdullah K.A., Reed W. (2018). 3D printing in medical imaging and healthcare services. J. Med. Radiat. Sci..

[59] Dodziuk H. (2016). Applications of 3D printing in healthcare. Kardiochir. Torakochirurgia Pol..

[60] Hurst E.J. (2016). 3D printing in healthcare: Emerging applications. J. Hosp. Librariansh..

[61] Perry A. (2018). 3D-printed apparel and 3D-printer: Exploring advantages, concerns, and purchases. Int. J. Fashion Des. Technol. Educ..

[62] Attaran M. (2017). The rise of 3-D printing: The advantages of additive manufacturing over traditional manufacturing. Bus. Horiz..

[63] Berman B. (2012). 3-D printing: The new Industrial Revolution. Bus. Horiz..

[64] Han T., Kundu S., Nag A., Xu Y. (2019). 3D printed sensors for biomedical applications: A review. Sensors.

[65] Mahmood M.A., Visan A.I., Ristoscu C., Mihailescu I.N. (2020). Artificial neural network algorithms for 3D printing. Materials.

[66] Elbadawi M., McCoubrey L.E., Gavins F.K., Ong J.J., Goyanes A., Gaisford S., Basit A.W. (2021). Disrupting 3D printing of medicines with machine learning. Trends Pharmacol. Sci..

[67] Karimi D., Dou H., Warfield S.K., Gholipour A. (2020). Deep learning with noisy labels: Exploring techniques and remedies in medical image analysis. Med. Image Anal..

[68] Yao J., Wang J., Tsang I.W., Zhang Y., Sun J., Zhang C., Zhang R. (2018). Deep learning from noisy image labels with quality embedding. IEEE Trans. Image Process.

[69] Dodge S., Karam L. (2016). Understanding How Image Quality Affects Deep Neural Networks Eighth International Conference on Quality of Multimedia Experience (QoMEX).

